# Promising clinical effect of arthroscopic autologous iliac bone grafting with suture anchor binding fixation for recurrent anterior shoulder instability

**DOI:** 10.3389/fsurg.2024.1398181

**Published:** 2024-09-19

**Authors:** Bo Tang, Peng Zhao, Ping Shi Wu, Cheng Fan

**Affiliations:** Sports Medicine Center, Xining First People’s Hospital, Qinghai, China

**Keywords:** glenoid defect, recurrent anterior shoulder dislocation, suture anchor, binding fixation, Eden–Hybinette, Latarjet

## Abstract

**Background:**

To evaluate the clinical efficacy of arthroscopic autologous iliac bone grafting with suture anchor binding fixation combined with a Bankart repair for recurrent anterior shoulder dislocation with a significant anterior glenoid defect.

**Methods:**

Patients with recurrent anterior shoulder dislocation with an anterior glenoid defect area greater than 20% admitted to our department from March 2019 to March 2022 were prospectively enrolled. Arthroscopic autologous iliac bone grafting with suture anchor binding fixation combined with a Bankart repair was performed. Computed tomography (CT) images were captured preoperatively, immediately after surgery, and at 3, 6, and 12 months postoperatively to evaluate the glenoid defect area, graft area, and graft healing. Shoulder function was assessed using the Instability Severity Index, Oxford Shoulder Instability, and Rowe scores recorded preoperatively and at the final follow-up. The shoulder range of motion, shoulder stability test, surgery-related complications, subluxation/dislocation, and revision surgery were also evaluated.

**Results:**

A total of 32 patients were included in the study, with an average follow-up time of 18.3 ± 6.3 months, when the graft healing rate was shown to be 100%. The area ratio of the graft to the glenoid was 37.6% ± 10.5% (range, 23.5%–44.1%) determined by an enface-view three-dimensional CT performed immediately after surgery, and 29.2 ± 8.2% (range, 19.6%–38.7%) at 12 months postoperatively. At the final follow-up, the glenoid defect had improved from 28.7 ± 6.4% (range, 20.5%–40.6%) before surgery to −10.2 ± 4.7% (range, −13.8% to 6.1%). The preoperative Rowe and Oxford scores were 56.4 ± 8.5 and 34.7 ± 7.1 respectively, which improved to 94.3 ± 6.7 and 15.3 ± 3.2 at the final follow-up (*p* < .001). All patients had no limited shoulder joint activity, no re-dislocation or revision surgery, and no neurovascular injury.

**Conclusions:**

For recurrent anterior shoulder dislocation with an anterior glenoid defect area greater than 20%, arthroscopic autologous iliac bone grafting with suture anchor fixation combined with a Bankart repair produced a promising clinical effect. A significant shoulder function score was achieved, as was a 100% bone healing rate and ideal glenoid reconstruction without major complications. Thus, this technique may be considered an alternative to the classic Latarjet approach to treat recurrent anterior shoulder dislocation with an anterior glenoid defect area greater than 20%.

**Level of Evidence:**

IV.

## 1 Introduction

Recurrent anterior dislocation of the shoulder joint is often accompanied by a certain degree of anterior glenoid bone defect. Griffith et al. found that 86% of patients with this condition had anterior glenoid bone defects revealed through computed tomography (CT) (1). In these patients, soft tissue procedures such as a Bankart repair often have a high risk of re-dislocation (2). In 2000, Burkhart and DeBeer firmly established bone loss as a contributor to the failure of arthroscopic Bankart repair for anterior glenohumeral instability in their classic article (3). Similarly, a significant decrease in stability with an osseous defect of 21% of the glenoid width was reported in a cadaveric study using sequential osteotomies of the anteroinferior glenoid (4). Therefore, a glenoid bone reconstruction operation is usually advocated in this context. Although there is no consensus on the critical bone defect area to carry out bone reconstruction, a more accepted standard is when the anterior glenoid bone defect area reaches 20% (5). A representative bone reconstruction can be conducted using the Latarjet technique, which has a high success rate but also has a high rate of complications reported in the literature. Hendy et al. retrospectively analyzed 190 cases of Latarjet surgery performed between August 2008 and July 2018 and reported a 9% (*n *= 15) complication rate in the 90 days after surgery and that 4.2% of patients required reoperation. Complications mainly included screw loosening or fracture (4.7%) and nerve injury (3.2%) (6). Compared with the Latarjet technique, iliac bone reconstruction has a long history. It was first introduced by Eden, who harvested an autologous tibial bone and gently transplanted it into the glenoid (7). Hybinette took an autologous iliac bone and sutured the articular capsule tightly above the bone to stabilize it (8). The technique was questioned for a long time because of the possible incidence of osteoarthritis (9, 10); however, later authors questioned whether osteoarthritis was present in these cases (11). A subsequent study confirmed that only 7 of 35 patients after Eden–Hybinette surgery developed stage 1 osteoarthritis during an average follow-up time of 9.2 years (12). In 2017, Giannakos et al. used the Eden–Hybinette technique to revise 12 cases of shoulder anterior instability after Latarjet surgery (10 cases of Latarjet, 2 cases of Bankart). During an average follow-up time of 28.8 months, 67% (*n *= 8) of the cases had satisfactory outcomes, and no cases required reoperation due to instability (13). Similarly, Boileau et al. used the Eden–Hybinette technique to revise seven cases of Latarjet failure. During an average follow-up time of 21 months, there were no nerve injuries or implant-related problems, and no patients required reoperation. All but one patient had satisfactory outcomes, with stable shoulder joints and constant scores that improved from 32 to 81 (14). Thus, the Eden–Hybinette technique has attracted attention again, and its clinical efficacy is readily comparable to the more popular Latarjet (15). At present, there are various ways to fix a transplanted bone block using the Eden–Hybinette technique, mainly including rigid screw fixation and non-rigid fixation of the suture button. Given that screw fixation also produces complications related to Latarjet surgery, such as screw fracture and displacement, non-rigid suture button fixation has been used in recent years with good clinical outcomes (16, 17). However, this technique requires the use of special guiding tools to drill bone tunnels. At our center, arthroscopic suture binding fixation of autologous iliac grafts, which can be performed with conventional arthroscopic shoulder tools, has been employed to manage these dislocations in recent years. We conducted the present study to evaluate clinical outcomes and graft healing. We hypothesized that this technique could produce a superior clinical effect and graft healing rates.

## 2 Materials and methods

### 2.1 Patients

This was a prospective case series study approved by the Ethics Committee of our hospital (Protocol number: 2021-M-19). All patients gave their informed consent prior to their inclusion in the study. We prospectively enrolled patients with recurrent anterior shoulder dislocation who were treated with arthroscopic autologous iliac bone grafting with suture anchor binding fixation combined with a Bankart repair from March 2019 to March 2022. The inclusion criteria were (1) patients with recurrent anterior shoulder dislocation and (2) a glenoid defect area >20%. The exclusion criteria were (1) a history of shoulder soft tissue or bone reconstruction surgery such as Latarjet or Bristow; (2) other lesions such as a rotator cuff tear or labrum injury; and (3) a history of epilepsy and other special conditions.

### 2.2 Surgical techniques

**Figure 1:**
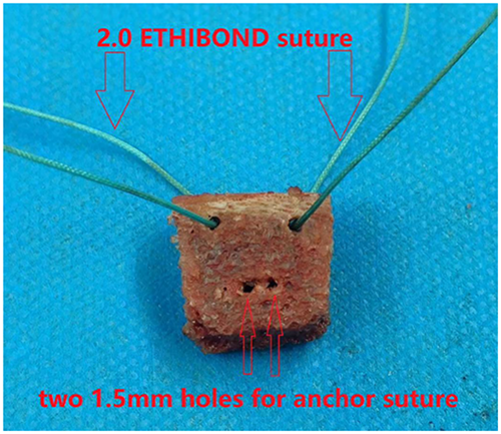
Bicortical iliac bone blocks were harvested. Two 2.0 mm bone tunnels were drilled at the left and right corners of the long axis of the bone block and a No. 2 ETHIBOND suture was inserted as a guide wire. Two 1.5 mm bone tunnels, 3 mm apart, were drilled 8 mm below the bone surface to pass through the suture of the medial anchor of the glenoid.

Taking the left shoulder as an example, the patient was in the lateral decubitus position after general anesthesia. First, a 2 cm long, approximately 8 mm wide, and 1.5 cm high autologous bicortical iliac bone block was harvested. The width of the bone mass refers to the width of the contralateral glenoid; that is, the width of the bone mass equals the affected glenoid width subtracted from the width of the contralateral glenoid (18). Two 2.0 mm bone holes approximately 3 mm below the bone surface were drilled at the left and right corners of the long axis of the bone block, and a No. 2 ETHIBOND suture was put into each hole as a guide. Then, two 1.5 mm bone holes approximately 3 mm apart were drilled 8 mm below the bone surface (Figure 1).

**Figure 2:**
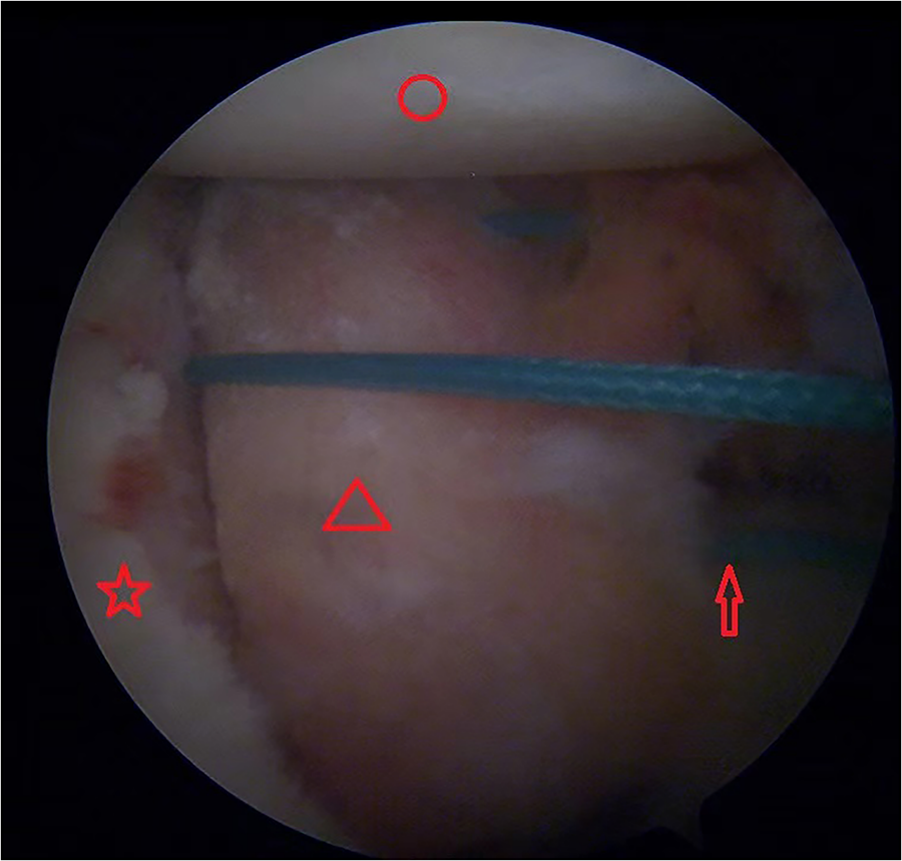
Left shoulder view from the anterosuperior portal. The iliac bone block was put into the glenohumeral joint along the two suture ends and passed through the two 1.5 mm holes. Red circle, humeral head; red pentagram, glenoid; red triangle, iliac bone graft; red arrow, anchor suture ends.

Second, standard posterior, anterior, and anterior–superior approaches were established, and the anterior capsule-labrum from the 5 to 11 o'clock position was fully released, and a 3 mm strip of bone bed on the anterior edge of the glenoid was created. Next, at the 8:30 position, a 2.9 mm absorbable suture anchor (Smith & Nephew, Gryphon) was inserted approximately 8 mm below the anterior glenoid bone surface. This distance was roughly measured with the tip of the arthroscope probe, which was 4 mm in length. The four tails of this suture anchor were pulled out and passed through the two 1.5 mm holes of the iliac bone block. The block was then routed into the glenohumeral joint through the rotator cuff interval, and two tails of the same color were tied to the surface of the iliac bone block to fix it in place (Figure 2).

**Figure 3:**
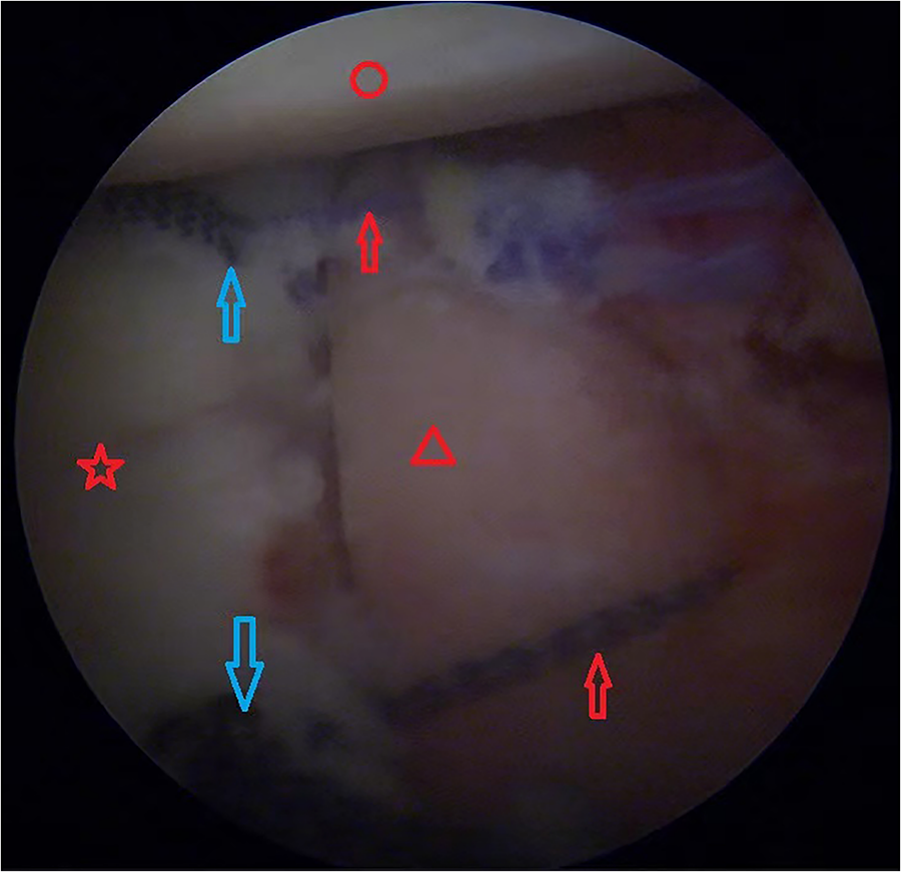
Left shoulder view from the anterosuperior portal. Two 2.9 mm anchors (blue arrows) were inserted into the glenoid, the suture ends were guided with 2.0 ETHIBOND suture through the 2.0 mm holes in the iliac bone block and then tied with another suture end. Red circle, humeral head; red pentagram, glenoid; red triangle, iliac bone graft; red arrow, tied anchor suture; blue arrows, two 2.9 mm anchors.

Two 2.9 mm suture anchors (Smith & Nephew, Gryphon) were then placed at the 7 and 9:30 positions on the anterior glenoid bone bed strip. One tail of each anchor was guided by the corresponding No. 2 ETHIBOND line of the iliac bone block to pass through the 2.0 mm hole, then tied with another tail on the surface of the iliac bone block; thus, the grafting iliac bone block was firmly tied up with the sutures of the three anchors (Figure 3).

**Figure 4:**
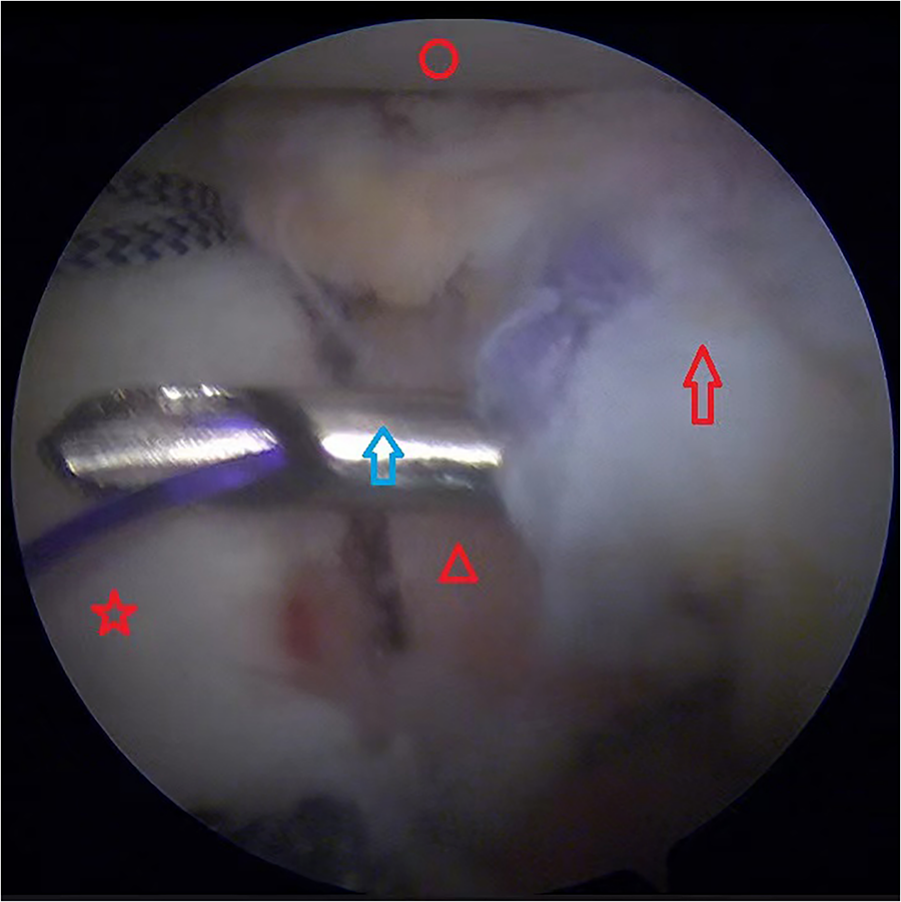
Left shoulder view from the anterosuperior portal. The iliac bone graft was covered by the labrum, which was sutured with the anchor sutures. Red circle, humeral head; Red pentagram, glenoid; red triangle, iliac bone graft; red arrow, labrum; blue arrow, the lasso passed through the labrum.

**Figure 5:**
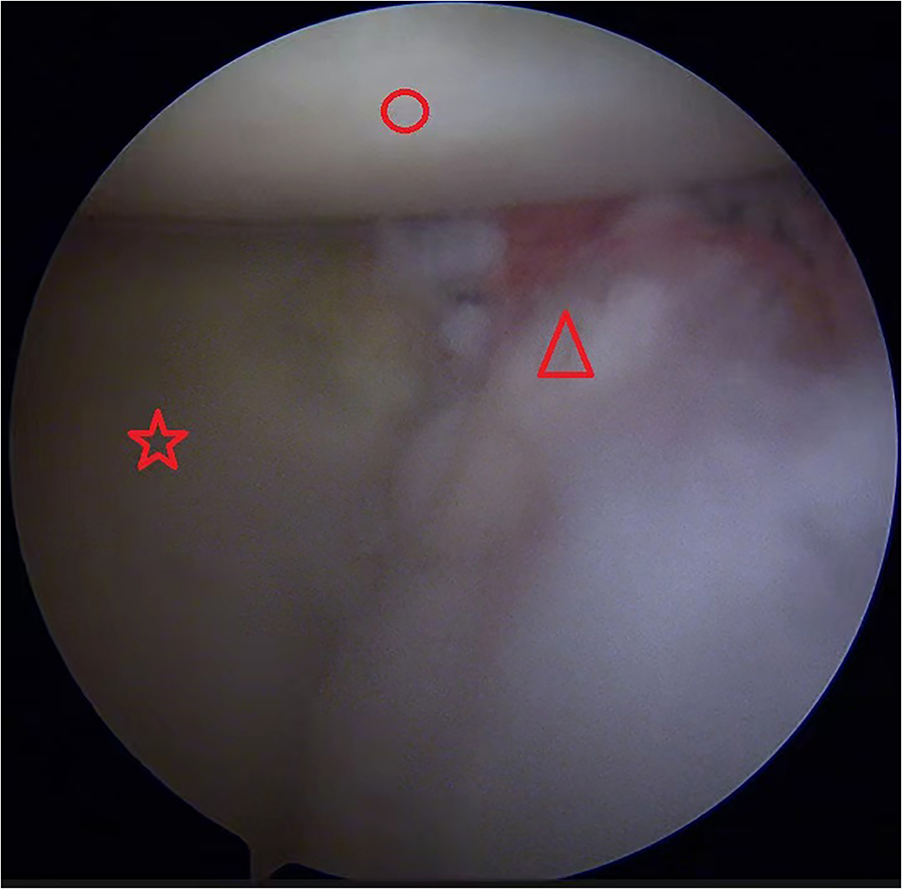
Left shoulder view from the anterosuperior portal. The labrum was sutured with the anchor sutures and then the iliac bone graft was completely covered. Red circle, humeral head; red pentagram, glenoid; red triangle, labrum.

The capsule-labrum tissue was then reduced to cover the iliac bone block. The remaining two sutures on the anterior glenoid bone bed were used to suture the capsule-labrum tissue in the corresponding position (Figure 4) and then tied, and the grafted iliac bone block was completely covered (Figure 5). The humeral side procedure (humeral bone grafting or Remplissage) depended on the engagement of the Hill–Sachs lesion after autologous iliac bone grafting, which was evaluated during the operation. In our study, we did not find off-track lesions after autologous iliac bone grafting, so the Hill–Sachs lesion was left untreated in this group of patients.

### 2.3 Postoperative rehabilitation

The shoulder was immobilized in a sling in the neutral external position immediately after surgery. During the first 3 weeks after surgery, limb suspension and pendulum exercises were performed on the affected shoulder. Later, active forward elevation, abduction, and external rotation with the assistance of the contralateral arm were allowed. Finally, 2 months after surgery, the sling was removed and 3 months after surgery muscle strengthening exercises were initiated.

### 2.4 Imaging evaluation

**Figure 6:**
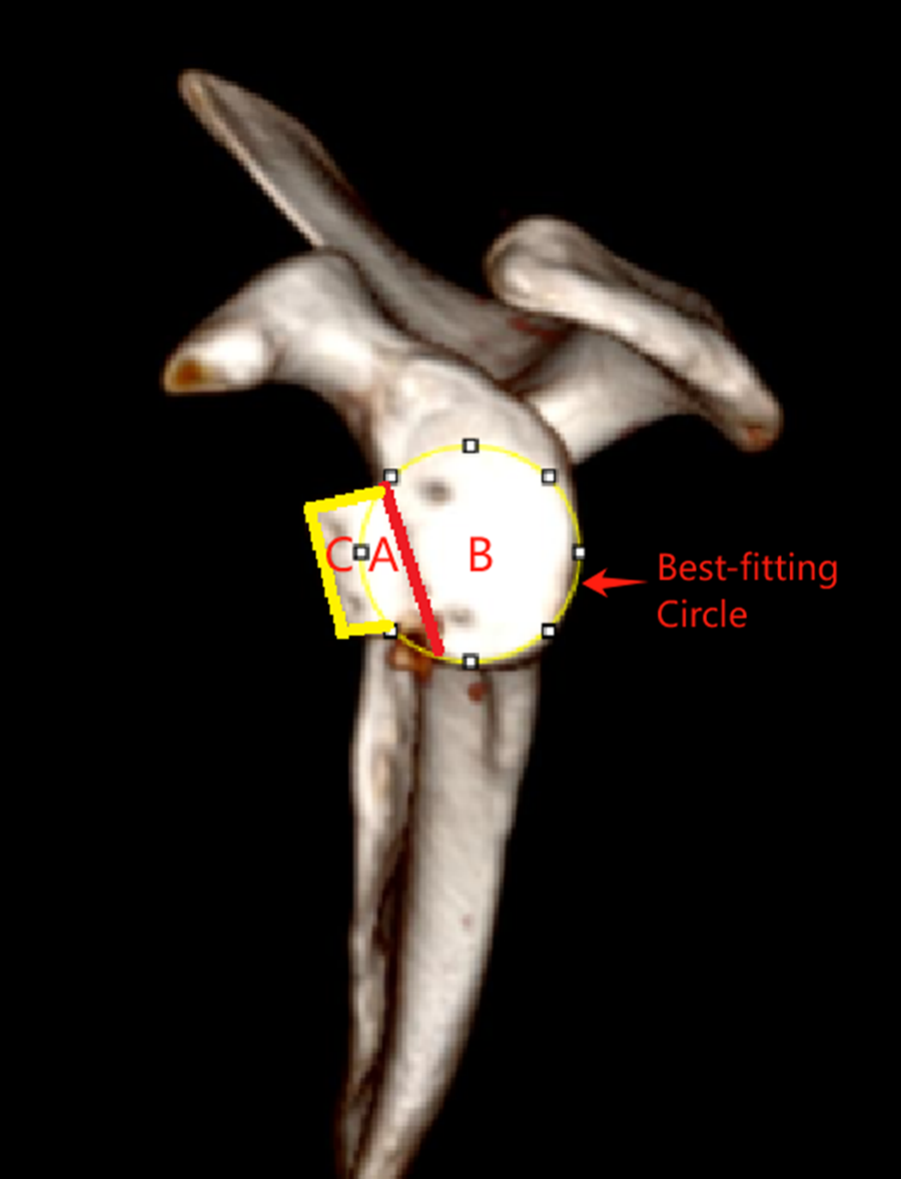
Measurement of the area of the glenoid. The glenoid defect area **(A)**. The remaining glenoid area within the best-fitting circle **(B)**. Enlarged glenoid area **(C)**.

CT scans and sagittal, coronal, and three-dimensional reconstructions of the shoulder glenoid were performed preoperatively and at 1 day and 3, 6, and 12 months postoperatively. The best-fitting circle technique was employed to measure the glenoid defect (19). First, three-dimensional reconstructions of the postoperative glenoid with subtraction of the humeral head were performed, and the best-fitting circles were placed based on the enface glenoid view. Second, the area of the glenoid bone, the iliac bone graft block, and the glenoid defect area within the best-fitting circle were measured with ImageJ software (National Institutes of Health, USA), which also permitted the ratio of the iliac bone graft block to the glenoid to be calculated. The grafting bone blocks outside the best-fitting circle were considered to be the enlarged area of the normal glenoid (Figure 6). The bone healing standard was the continuous bone cortex between the iliac bone graft block and the glenoid on the axial CT plane.

### 2.5 Postoperative follow-up

All patients were regularly followed up for 3 weeks, and then for 3, 6, and 12 months after surgery. Pain management and rehabilitation guidance were mainly carried out within 3 months of follow-up. The Rowe and Oxford scores were used as indices of the function of the shoulder at the last follow-up when the final shoulder stability test and range of motion were evaluated. The assessment of the shoulder range of motion mainly included forward flexion (FF), internal rotation (IR), passive external rotation at 0° of abduction (ER1) and 90° of abduction (ER2), and passive abduction with the examiner stabilizing the scapula (AB). Internal rotation was scored based on the highest vertebral level reached by the hand of the affected side: buttock, 2 points; sacrum, 4 points; 3rd lumbar vertebra, 6 points; 12th thoracic vertebra, 8 points; and the 7th thoracic vertebra, 10 points. All complications detected during the follow-up period were recorded, including shoulder adhesion, fixation failure, non-union, positive shoulder stability fear test, and re-dislocation of the shoulder joint. Revision surgery for any reason except trauma was considered to be a surgical failure.

### 2.6 Statistical analysis

All statistical analyses were performed using SPSS version 19.0. Continuous variables were expressed as the mean ± standard deviation and a matched *t*-test was employed for comparisons of these continuous variables before and after the treatment. Rates were expressed as percentiles, and *p-*values ≤0.05 were considered to be statistically significant.

## 3 Results

### 3.1 Basic information on included patients

According to the inclusion criteria, we prospectively included 33 patients with recurrent anterior shoulder dislocation who were treated with arthroscopic autogenous iliac bone grafting with suture anchor binding fixation combined with a Bankart repair from March 2019 to March 2022. One patient refused postoperative follow-up, and the remaining 32 patients completed all relevant follow-up assessments within 1 year and on schedule. The included cohort was comprised of 25 men and 7 women, with an average age of 22.6 ± 5.7 years and an average follow-up time of 18.3 ± 6.3 months. All shoulder injuries were unilateral shoulder dislocations, and the dislocated shoulder was the dominant shoulder in 24 cases. The Hill–Sachs lesion was found in all cases. The average instability severity index score (ISIS) was 4.4 ± 2.1 points. The average number of shoulder dislocations was 9.8 ± 6.7 times, and the interval from the initial dislocation to the operation was 15.6 ± 8.4 months (Table 1).

**Table 1:** General information on the included patients.

Variable	
Age (years)	22.6 ± 5.7
Gender	Men 25; Women 7
Affected dominant shoulder (*n*)	24
Number of dislocations (*n*)	9.8 ± 6.7
Time from first dislocation to surgery (months)	15.6 ± 8.4
ISIS score	4.4 ± 2.1
Beighton score	3.6
Follow-up (months)	18.3 ± 6.3

### 3.2 Grafted iliac bone healing rate

All the grafted bone blocks healed, with 27 cases showing healing on follow-up CT at 6 months postoperatively and 5 cases showing healing on follow-up CT at 3 months postoperatively.

### 3.3 Glenoid bone defect reconstruction

Among the 32 patients included, the preoperative evaluation of glenoid bone defect was 28.7 ± 6.4% (range, 20.5%–40.6%) and the postoperative glenoid bone defect was −10.2 ± 4.7% (range, −13.8% to 6.1%). The area ratio of the grafted bone block to the preoperative glenoid as determined by CT immediately after surgery was 37.6 ± 10.5% (range, 23.5%–44.1%), and the area ratio was 29.2 ± 8.2% (range, 19.6%–38.7%), indicating a certain degree of bone remodeling and bone resorption.

### 3.4 Rowe and Oxford scores

Compared with the preoperative values, the average Rowe and Oxford scores of all patients were significantly improved. The preoperative Rowe score for all patients was 56.4 ± 8.5 points, which improved to 94.3 ± 6.7 points at the last follow-up (average 1 year) (*p* < .001). The preoperative Oxford score for all patients was 34.7 ± 7.1 points, which improved to 15.3 ± 3.2 points at the last follow-up (average 1 year) (*p* < .001) (Table 2).

**Table 2:** Comparison of preoperative and postoperative Rowe and Oxford scores of patients.

	Rowe score	Oxford score
Preoperatively	56.4 ± 8.5	34.7 ± 7.1
Final follow-up	94.3 ± 6.7^a^	15.3 ± 3.2^a^

^a^ Significant difference between preoperative and postoperative Rowe and Oxford scores (*p* < 0.001).

### 3.5 Comparison of shoulder range of motion

At the last follow-up, no patient exhibited a significant loss of shoulder range of motion (Table 3).

**Table 3:** Comparison of shoulder joint ranges of motion.

	FF	IR	AB	ER (1)	ER (2)
Preoperatively	176.3	9.2	100.4	69.6	77.5
Final follow-up	177.1	9.6	99.8	69.2	76.8

### 3.6 Complications

No patient had major complications such as nerve or vascular injury during the perioperative period. At follow-up, all patients had good wound healing. Four patients still had significantly limited shoulder mobility 3 months after surgery, and were diagnosed with post-traumatic adhesive shoulder bursitis, which was cured with physiotherapy. Two patients had a positive shoulder stability fear test 1 year after surgery, but no dislocation occurred, and no further treatment was necessary. Two patients still had pain at the iliac bone harvesting site at the 6-month follow-up, and oral non-steroidal anti-inflammatory drugs were prescribed. The pain had disappeared at the last follow-up.

## 4 Discussion

The most important finding of the present study was that arthroscopic autologous iliac bone grafting with suture anchor binding fixation can effectively treat recurrent anterior shoulder dislocation with a glenoid defect area >20% and a graft healing rate of 100%. In recent years, increasing attention has been paid to iliac bone grafting and non-rigid fixation. In 2020, Malahias et al. systematically reviewed the treatment of anterior shoulder dislocation with non-rigid fixation of bone graft (20). A total of eight studies were included, five of which used suture button fixation, one of which used suture anchor fixation, and two of which used J-shaped iliac bone compression fixation without internal fixation. The authors found that non-rigid fixation technology produced reliable clinical outcomes and satisfactory function for recurrent anterior shoulder instability with obvious glenoid defects, results consistent with ours. However, the latter authors found an overall non-union rate of 5.4% for the graft, especially the study by Gendre et al. (21) included in the systematic review, which reported an overall non-union rate of 17% (12/70). We believe that this rate was relatively high. The authors mentioned that all non-union cases were patients who had a history of smoking, which cannot be determined as a risk factor for non-union. Similarly, in the study by Bonnevialle et al. (22), the authors treated 88 cases of recurrent anterior shoulder instability with obvious glenoid defects with arthroscopic Latarjet double-button fixation. Four cases had early displacement of the graft, which the authors attributed to possible technical immaturity. However, we speculate that when the autologous coracoid process combined with the conjoint tendon were transferred to the anterior glenoid, non-rigid Button fixation may require more conservative rehabilitation schemes to avoid conjoint tendon traction on the graft and thus affect healing. Zhao et al. reported a 100% graft healing rate using allogeneic iliac bone non-rigid fixation (23). In our study, the graft healing rate was also 100%, which may suggest that conjoint tendon traction on the graft is a risk factor for non-union; however, robust evidence is still needed to support this conjecture.

The current mainstream interventions for anterior shoulder dislocation with glenoid defects are Latarjet and iliac bone grafting. A classic randomized controlled study was conducted by Moroder et al. in 2019, in which the authors compared the clinical outcomes of open Latarjet and iliac bone grafting for the treatment of recurrent anterior shoulder instability with obvious glenoid defects. The authors found no significant differences in relevant clinical scores, shoulder joint activity, etc. The Latarjet group had significant limitations of internal rotation, while 27% of the cases in the iliac bone grafting group had pain at the bone harvesting site (24). According to current literature reports, Latarjet produces higher complication rates and requires a greater learning curve. Cho et al. recently systematically reviewed the complications related to Latarjet surgery (25). The authors analyzed 35 articles with 2,560 Latarjet surgery cases (2,532 patients), and the overall complication rate was 16.1% (*n *= 412). Intraoperative complications included neurovascular injury, screw-related problems, fractures, and arthroscopic conversion to open surgery. Postoperative complications mainly included non-union of bone fragments. These findings support our previous speculation that conjoint tendon traction may be a risk factor for non-union of the grafted bone block. The iliac bone grafting technique is relatively simple, safe, and produces no major complications. The main problem is pain at the bone harvesting site, which has been reported often in the literature. In our study, two patients experienced pain at the bone harvesting site at the 6-month operation follow-up, but the pain disappeared at the final follow-up after being managed with oral non-steroidal anti-inflammatory drugs. Allogeneic iliac bone or an artificial bone block may be a solution to this problem. Taverna et al. treated 26 cases of recurrent anterior shoulder instability with glenoid bone defects by using non-rigid allogeneic iliac bone fixation. During the follow-up period of at least 2 years, the clinical outcomes were excellent, with no postoperative dislocation and a bone healing rate of 92.3% (26). Similarly, Zhao et al. reported a 100% bone healing rate by using allogeneic iliac bone suture anchor fixation. These findings confirmed the effectiveness and safety of allogeneic bone to some extent. However, limited sources of allogeneic bone may exist in many hospitals.

Currently reported non-rigid fixation methods mainly include Endobutton (Smith & Nephew, Andover, MA, USA), suture anchor, and J-type compression fixation, but our technique was different from these. Endobutton requires a special guiding tool to drill a tunnel, which may be unavailable in some hospitals. The bone block is prepared into a special J-type, and a matched slot in the anterior glenoid is also prepared in the J-type compression fixation technique; the bone block is routed into the bone slot by special tools and mounted in the bone slot. This series of operations presents certain difficulties, and there is a risk of bone fragmentation in patients with poor bone quality. The suture anchor method of Zhao et al. was similar to ours. They used the suture on the anchor to tie a knot directly to fix the bone block, but the bone block still floats unstably and can move up and down after knot fixation. Our technique was slightly different. An additional anchor was implanted approximately 8 mm below the glenoid surface. Two sutures were directly passed through the bone block and a knot was tied. The sutures of two additional anchors on the surface of the glenoid were passed through the upper and lower sides of the bone block, respectively, and a knot was tied. In this way, a three-point, triangular stable fixation was formed. These surgical operations were quite simple, and no special tools were required. Although there have been no biomechanical studies to compare it to screw fixation and Endobutton fixation, 100% graft healing without obvious displacement in our study confirms the reliability of this technique to a great extent.

It is worth mentioning that the postoperative patients in this study had almost no restriction of shoulder joint functions, and there was no obvious loss of range of motion compared with the preoperative range. We believe that this finding was due to the anatomical repair without splitting the subscapularis muscle, and the bone block was implanted through the rotator cuff interval without interference with the normal anatomical structure.

Physicians have a professional duty to provide the patient with all appropriate information about the potential risks, benefits, and specific advantages of one procedure vs. the other (27).Compared with the classic Latarjet operation, our technique has its potential risks and advantages. The potential risks mainly include pain at the bone harvesting site and non-union, and this is a new technique with short follow-up times, some unknown complications may occur over a longer follow-up period. This technique also shows obvious advantages, including simple operation without special tools, anatomical repair without splitting the subscapularis muscle, and absorbable suture anchors without screw-related problems. Regardless, the final treatment plan should be decided upon by the physician and the patient after a thorough discussion of the surgical benefits and risks.

This study had a number of obvious limitations. First, there was no control, such as some non-rigid and rigid fixation methods that have been reported so far. Second, there was no basic biomechanical research to confirm the biomechanical strength of our fixation technology. Further, relevant randomized controlled trials and basic biomechanical research will deepen the understanding of this technology. Third, the patient cohort was relatively small. As a single-center study, there were not many cases of anterior dislocation of the shoulder joint with obvious bone defects. Therefore, further multi-center, large-sample, prospective randomized controlled trials are needed in the future to conduct an in-depth evaluation of the clinical efficacy and safety of this technology.

## 5 Conclusions

For recurrent anterior shoulder dislocation with an anterior glenoid defect area >20%, arthroscopic autologous iliac bone grafting with suture anchor fixation combined with a Bankart repair produced promising clinical outcomes, achieving significant shoulder function scores, a 100% bone healing rate, and ideal glenoid reconstruction without major complications. Thus, this technique may be considered an alternative to the classic Latarjet procedure.

## Data Availability

The raw data supporting the conclusions of this article will be made available by the authors, without undue reservation.

## References

[1] Griffith JF Antonio GE Yung PS Wong EM Yu AB Ahuja AT Prevalence, pattern, and spectrum of glenoid bone loss in anterior shoulder dislocation: CT analysis of 218 patients. AJR Am J Roentgenol. (2008) 190(5):1247–54. 10.2214/AJR.07.3009 18430839

[2] Delgado C Calvo E Martínez-Catalán N Valencia M Luengo-Alonso G Calvo E. High long-term failure rates after arthroscopic Bankart repair in younger patients with recurrent shoulder dislocations: a plea for early treatment. Knee Surg Sports Traumatol Arthrosc. (2024). 10.1002/ksa.12391. [Epub ahead of print] 39101229

[3] Burkhart SS De Beer JF. Traumatic glenohumeral bone defects and their relationship to failure of arthroscopic Bankart repairs: significance of the inverted-pear glenoid and the humeral engaging Hill-Sachs lesion. Arthroscopy. (2000) 16(7):677–94. 10.1053/jars.2000.17715 11027751

[4] Itoi E Lee SB Berglund LJ Berge LL An KN. The effect of a glenoid defect on anteroinferior stability of the shoulder after Bankart repair: a cadaveric study. J Bone Joint Surg Am. (2000) 82(1):35–46. 10.2106/00004623-200001000-00005 10653082

[5] Provencher MT Midtgaard KS Owens BD Tokish JM. Diagnosis and management of traumatic anterior shoulder instability. J Am Acad Orthop Surg. (2021) 29(2):e51–61. 10.5435/JAAOS-D-20-00202 33275397

[6] Hendy BA Padegimas EM Kane L Harper T Abboud JA Lazarus MD Early postoperative complications after Latarjet procedure: a single-institution experience over 10 years. J Shoulder Elbow Surg. (2021) 30(6):e300–8. 10.1016/j.jse.2020.09.002 33010440

[7] Eden R. Zur operation der habituellen schulterluxation unter mitteilung eines neuen verfahrens bei abriß am inneren pfannenrande. Dtsch Z Chir. (1918) 144:269–80. 10.1007/BF02803861

[8] Hybbinette S. De la transplantation d’un fragment osseux pour remédier aux luxations récidivantes de l’épaule: constatations et résultats opératoires. Acta Chir Scand. (1932) 71:26.

[9] Rachbauer F Ogon M Wimmer C Sterzinger W Huter B. Glenohumeral osteoarthrosis after the Eden-Hybbinette procedure. Clin Orthop Relat Res. (2000) 373:135–40. 10.1097/00003086-200004000-00016 10810470

[10] Wildner M Wimmer B Reichelt A. Osteoarthritis after the Eden-Hybbinette-Lange procedure for anterior dislocation of the shoulder. A 15 year follow up. Int Orthop. (1994) 18(5):280–3. 10.1007/BF00180226 7852006

[11] Willems WJ. Reconstruction of glenoid bone defects in shoulder instability with autologous bone. Curr Rev Musculoskelet Med. (2014) 7(1):12–5. 10.1007/s12178-013-9200-0 24470115 PMC4094119

[12] Steffen V Hertel R. Rim reconstruction with autogenous iliac crest for anterior glenoid deficiency: forty-three instability cases followed for 5–19 years. J Shoulder Elbow Surg. (2013) 22(4):550–9. 10.1016/j.jse.2012.05.038 22947237

[13] Giannakos A Vezeridis PS Schwartz DG Jany R Lafosse L. All-arthroscopic revision Eden-Hybinette procedure for failed instability surgery: technique and preliminary results. Arthroscopy. (2017) 33(1):39–48. 10.1016/j.arthro.2016.05.021 27432589

[14] Boileau P Duysens C Saliken D Lemmex DB Bonnevialle N. All-arthroscopic, guided Eden-Hybbinette procedure using suture-button fixation for revision of failed Latarjet. J Shoulder Elbow Surg. (2019) 28(11):e377–88. 10.1016/j.jse.2019.03.022 31331667

[15] Villatte G Spurr S Broden C Martins A Emery R Reilly P. The Eden-Hybbinette procedure is one hundred years old! A historical view of the concept and its evolutions. Int Orthop. (2018) 42(10):2491–5. 10.1007/s00264-018-3970-3 29744648

[16] Kalogrianitis S Tsouparopoulos V. Arthroscopic iliac crest bone block for reconstruction of the glenoid: a fixation technique using an adjustable-length loop cortical suspensory fixation device. Arthrosc Tech. (2016) 5(6):e1197–202. 10.1016/j.eats.2016.07.007 28149713 PMC5262518

[17] Avramidis G Kokkineli S Trellopoulos A Tsiogka A Natsika M Brilakis E Excellent clinical and radiological midterm outcomes for the management of recurrent anterior shoulder instability by all-arthroscopic modified Eden-Hybinette procedure using iliac crest autograft and double-pair button fixation system: 3-year clinical case series with no loss to follow-up. Arthroscopy. (2021) 37(3):795–803. 10.1016/j.arthro.2020.10.036 33127552

[18] Griffith JF. Measuring glenoid and humeral bone loss in shoulder dislocation. Quant Imaging Med Surg. (2019) 9(2):134–43. 10.21037/qims.2019.01.06 30976536 PMC6414777

[19] Sugaya H Moriishi J Dohi M Kon Y Tsuchiya A. Glenoid rim morphology in recurrent anterior glenohumeral instability. J Bone Joint Surg Am. (2003) 85(5):878–84. 10.2106/00004623-200305000-00016 12728039

[20] Malahias MA Mitrogiannis L Gerogiannis D Chronopoulos E Kaseta MK Antonogiannakis E. Non-rigid fixation of the glenoid bone block for patients with recurrent anterior instability and major glenoid bone loss: a systematic review. Shoulder Elbow. (2021) 13(2):168–80. 10.1177/1758573219872512 33897848 PMC8039760

[21] Gendre P Thélu CE d'Ollonne T Trojani C Gonzalez JF Boileau P. Coracoid bone block fixation with cortical buttons: an alternative to screw fixation? Orthop Traumatol Surg Res. (2016) 102(8):983–7. 10.1016/j.otsr.2016.06.016 27720375

[22] Bonnevialle N Thélu CE Bouju Y Vogels J Agout C Duriez P Arthroscopic Latarjet procedure with double-button fixation: short-term complications and learning curve analysis. J Shoulder Elbow Surg. (2018) 27(6):e189–95. 10.1016/j.jse.2017.12.007 29337029

[23] Zhao J Huangfu X Yang X Xie G Xu C. Arthroscopic glenoid bone grafting with nonrigid fixation for anterior shoulder instability: 52 patients with 2- to 5-year follow-up. Am J Sports Med. (2014) 42(4):831–9. 10.1177/0363546513519227 24510068

[24] Moroder P Schulz E Wierer G Auffarth A Habermeyer P Resch H Neer Award 2019: Latarjet procedure vs. Iliac crest bone graft transfer for treatment of anterior shoulder instability with glenoid bone loss: a prospective randomized trial. J Shoulder Elbow Surg. (2019) 28(7):1298–307. 10.1016/j.jse.2019.03.035 31129017

[25] Cho CH Na SS Choi BC Kim DH. Complications related to Latarjet shoulder stabilization: a systematic review. Am J Sports Med. (2023) 51(1):263–70. 10.1177/03635465211042314 34633879

[26] Taverna E Garavaglia G Perfetti C Ufenast H Sconfienza LM Guarrella V. An arthroscopic bone block procedure is effective in restoring stability, allowing return to sports in cases of glenohumeral instability with glenoid bone deficiency. Knee Surg Sports Traumatol Arthrosc. (2018) 26(12):3780–7. 10.1007/s00167-018-4921-7 29623353

[27] Bolcato V Franzetti C Fassina G Basile G Martinez RM Tronconi LP. Comparative study on informed consent regulation in health care among Italy, France, United Kingdom, Nordic countries, Germany, and Spain. J Forensic Leg Med. (2024) 103:102674. 10.1016/j.jflm.2024.102674 38502996

